# The European Working Time Directive: Will Modern Surgical Training in the United Kingdom Be Sufficient?

**DOI:** 10.7759/cureus.21797

**Published:** 2022-01-31

**Authors:** Matthew G Wyman, Roy Huynh, Corinne Owers

**Affiliations:** 1 Department of General Surgery, Sheffield Teaching Hospitals NHS Foundation Trust, Sheffield, GBR; 2 Faculty of Medicine, University of New South Wales, Sydney, AUS; 3 Department of General Surgery, Blackpool Teaching Hospitals NHS Foundation Trust, Blackpool, GBR

**Keywords:** work schedule, general surgery, qualitative research, education in general surgical residency, european working time directive, post grad medical education

## Abstract

Purpose

The introduction of the European Working Time Directive in 2009 limits doctors in the United Kingdom to a 48-hour working week. The reduction in surgical training time raises concern over the ability of future surgeons to deliver safe and effective care.

Methods

This interview-based qualitative study was conducted within a tertiary referral centre in the United Kingdom. Nine consultant general surgeons were interviewed with the aim of investigating how surgical trainees can comply with the European Working Time Directive whilst gaining sufficient knowledge, skill and experience to be safe surgeons.

Results

Consultants felt that the European Working Time Directive has impacted surgical training, patient care, service provision, and the professional attitudes of trainees. They felt that current surgical trainees have a relative lack of experience compared to previous generations, which has impacted their ability to manage complex patients. The consultant-trainee relationship was felt to have suffered due to shorter working hours. Furthermore, the move towards shift work has resulted in a lack of continuity of care for patients. Consultants suggested reconfiguring theatre lists to maximise opportunities for trainees. They also recommended that trainees seek out alternative learning methodologies such as simulation, and consider clinical fellowships at the completion of their training to maximise their experience and surgical skills prior to consultancy.

Conclusion

This study highlights the concerns that senior surgeons working in a busy tertiary referral centre have towards the European Working Time Directive and modern surgical training. The authors recommend that both trainees and consultants have a responsibility to maximize opportunities during training, and that mentorship will need to continue at the consultant level. Further research in other centres can determine whether these sentiments are widespread, and whether institutional steps should be taken to change the way that modern surgeons are trained.

## Introduction

The European Working Time Directive (EWTD) provides legislation for employers regarding minimum health and safety requirements for employee working hours, time off, breaks, and minimum rest periods. The EWTD was fully implemented for doctors in the United Kingdom in 2009 and mandates that junior doctors are unable to work more than a 48 hour week when averaged over a 26-week period, or a maximum of 56 hours after signing an opt-out clause [1]. It is unclear whether the strict limits stipulated by the EWTD have improved patient safety. There is evidence that shorter and fewer working hours results in fewer medical errors being made [2]. However, there is also evidence that the increased frequency of handovers and staffing changes limits continuity of care, and increases the likelihood of medical errors [3]. Professional associations such as the British Medical Association (BMA), the General Medical Council (GMC), and the Association of Surgeons in Training (ASiT) have argued that modern medical and surgical trainees are exposed to fewer cases, gain less clinical knowledge, and have less ability to perform complex procedures. The Accreditation Council for Graduate Medical Education (ACGME) demonstrated a significant decrease in the number of operations performed by surgical trainees between 2001 and 2006, partly due to the requirement for trainees to spend time out of hours when no elective operating occurs [4]. In specialties such as general surgery, which rely upon high levels of specialist knowledge and technical skill, the impact of the EWTD on training could therefore be substantial [5].

Over the next few years, the National Health Service (NHS) will see an influx of newly appointed consultants without the experience of their predecessors; furthermore, given how drastically the COVID-19 pandemic has impacted surgical training, it is essential to explore issues surrounding training and service provision. Although numerous quantitative studies have been conducted to investigate the concerns of surgeons and surgical trainees about the amount of time they spend in training, few qualitative studies have explored the impact of working time regulations on surgical training [6], and to our knowledge none have explored the opinions of senior surgical consultants. This study aims to explore the opinions of consultant surgeons appointed prior to the introduction of the 2009 EWTD, in order to investigate how they perceive the EWTD to have impacted upon training, and how they believe current surgical trainees can work within the confines of the EWTD to achieve the knowledge and skills required as a modern general surgeon.

## Materials and methods

Study design

This qualitative, interview-based study was conducted in 2015. This study was reviewed and approved by the University of Dundee ethics committee (UREC 14087). All participants provided written informed consent.

Identification and recruitment of participants

Participants included consultant surgeons working within the general surgery directorate at Sheffield Teaching Hospitals NHS Foundation Trust, who had attained their Certificate of Completion of training (CCT) before the full introduction of the EWTD. A purposive sampling technique [7] was used to select participants from each general surgery sub-specialty (upper gastrointestinal (GI), hepatobiliary, endocrine/breast, colorectal, and renal transplant).

Each interview was semi-structured; the researcher in each case asked certain specific questions, but otherwise the content of the discussion was participant-led and exploratory. All interviews were recorded using an OLYMPUS DS650 audio recorder. Each interview was transcribed verbatim by a researcher (CO) and then checked for accuracy. Anonymized paper copies of interviews were kept in a locked file in a secured office. Recruitment/interviews continued until data saturation was reached, or a maximum of 20 participants had been interviewed. The first study interview was conducted in May 2015. No new themes emerged by the ninth interview, conducted in August 2015, and accordingly no further interviews took place. Participants were coded in order of the interviews - the first consultant interviewed was coded as ‘P1’, and the final consultant was coded as ‘P9’. Table 1 illustrates participant demographics.

**Table 1:** Demographic data of general surgery consultants participating in study interviews (n=9)

Sex	Ethnicity	Specialty	Consultant start date
Male - 8	Caucasian - 7	Colorectal - 4	1995-1999 - 2
Female -1	Asian - 2	Upper GI - 2	2000-2004 - 3
		Endocrine - 1	2005-2009 - 4
		Hepatobiliary - 1	
		Renal transplant - 1	

Analysis

Analysis was conducted using NVIVO for MAC Version 10.0.4 (1066). General inductive data analysis was used to categorize and code the data from the interviews [8]. Specific small segments of text from each transcript that were deemed relevant to the study objectives were identified and categorised. Each time a segment of text was found which did not relate to any of the existing categories, a new category was created and all the previous transcripts were re-read to see if any text from these was relevant to the new category. This process continued until no new categories have been created, indicating that data saturation had been reached. Similar categories were clustered into “themes”, which are explored below.

## Results

**Figure 1:**
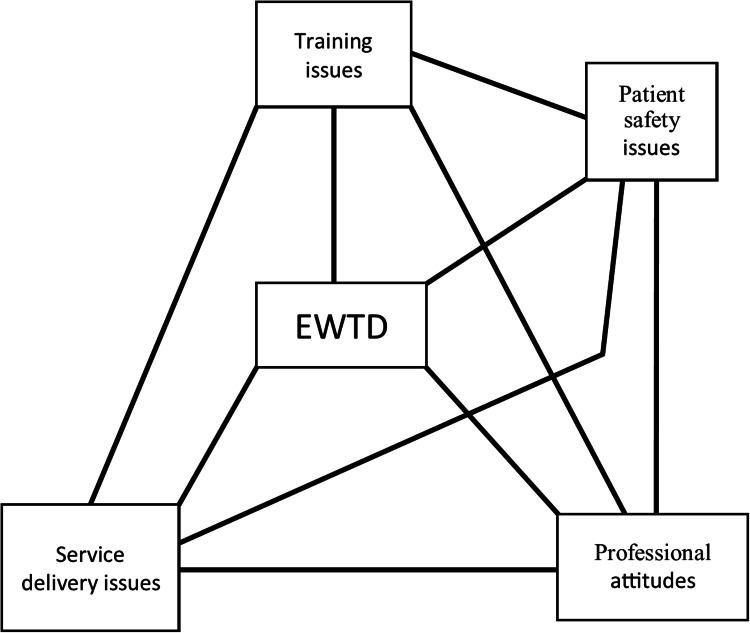
Emergent themes from interviews of nine general surgery consultants about their opinions of the impact of the European Working Time Directive (EWTD) on surgical training, and how each theme links to related themes

Analysis of transcription yielded four main themes: training issues, service issues, patient issues, and professional attitudes. Each theme was, from the perspective of the participants, directly related to the others - for instance, a perceived lack of training may affect the professional attitude of trainees, which may impact upon the service delivered to patients, which in turn, may raise patient safety issues (Figure 1). Although themes were interconnected, for clarity each will be discussed separately.

Training issues

By the time a trainee achieves their CCT and becomes a consultant, they would be expected to independently run outpatient clinics, perform endoscopies, and undertake straightforward operations. Study participants unanimously felt that current trainees had a relative lack of experience compared to themselves at the same milestone, due to the EWTD.

“Once it phased in and came to the 48-hour rule, it has shrunk the opportunity for the surgical trainee to have hands on surgical training by over 50%.” (P9)

Some consultants noted that surgery, as a craft skill, required the practical acquisition of knowledge and skills.

“It (the EWTD) doesn't necessarily detract from knowledge, because that can be gained from a book, but it certainly does detract from the experience in theatre… which is what surgery is all about.” (P1)

The diminished experience of modern trainees impairs their ability to operate and manage complex patients - which is a concern due to the increasingly complex disease processes, evidence-based practices, and operative techniques which modern surgeons must master.

Consultants were concerned about the loss of the traditional ‘firm’ structure within surgical departments. It was this firm structure that helped professional relationships to develop as consultant and trainee became aware of each other’s abilities. By contrast, many current consultants felt that they rarely see their trainees, impacting their ability to develop a good training relationship.

“It’s lowered my expectations of the trainees that are coming towards us because they’re not around as much. I know that’s not their fault - it's the nature of how things have changed.” (P6)

Consultants were asked what they believed current trainees could do to improve the quality of their own training, and hence improve competency and experience. Most consultants felt that the hours of work should be increased to gain experience:

“Opt-out of the EWTD so they can work 56 hours a week - that would mean fewer zero-days, which would mean more exposure.” (P8)

It was recognized that despite the EWTD, many trainees still worked outside of their designated hours, leading to a culture of “having to go above and beyond”.

“If a trainee wants to come in and do extra for their learning benefit, then… I’ll support them in that.” (P1)

Post-CCT clinical fellowships with limited on-call commitments were commonly cited as opportunities for trainees to obtain experience prior to their appointment as a consultant.

“I’d do a fellowship. I think whatever you’re doing, it enhances your experience, and I think you’ll be a better consultant.” (P5)

Simulation and skills training are becoming more utilised within surgical education. Laparoscopic and endoscopic simulators are available in many hospitals, and trainees can learn how to perform the basic steps of many different operations.

“I think we need to make use of the technology that is out there - simulation, mannequins, laparoscopy, cadavers - not courses, but for people to train on a regular basis. The virtual reality simulators that are out there are amazing tools, and I think we need to spend the money to get trainees on them.” (P7)

It was also noted by many of the consultants that they as trainers need to take some responsibility for improving training.

“When we’re planning the week… what we currently do is we populate the operating lists first, rather than have a balanced week for trainees. I think we should have a more balanced and realistic approach to what you need for your training, rather than just operating and operating.” (P8)

Conversely, some consultants felt that the increased level of supervision introduced as a result of the EWTD has resulted in trainees developing surgical techniques and skills more safely, rather than “learning by mistakes”.

“I think the advantage to training now is that you’re supervised more closely, you get better training and I think you will make fewer mistakes because your consultants have made the mistakes and they will prevent you from making the mistakes.” (P5)

Service delivery issues

Consultants believed that service provision and training were inextricably linked. As current trainees become consultants, there were concerns that they would be less experienced, which would have a knock-on effect for future trainees and the NHS.

“Certainly looking at some of the trainees coming through, I wouldn't like to think they were operating on me in 20 years time.” (P1)

Some of the consultants talked about how the workload of the NHS seems to be slowly moving up the hierarchy, with seniors doing more of the work that they used to do as trainees.

“You've reduced the hours that the service can be provided for and reduced the number of people, therefore someone else is doing more work. And the people doing this extra work are the consultants.” (P6)

“So how has my attitude changed? My expectations are lower: (as a consultant) on call, I expect to be in a lot. During my last 18 months as a registrar I had the boss in once.” (P3)

Consultants talked about the difficulties in trying to co-ordinate service provision, training, and the limited number of hours that doctors are allowed to work. It was noted that providing training made it more difficult to provide an effective service:

“I think you’re being trained within a system that is not designed for training but for providing a service.” (P6)

It was noted that even if the EWTD restrictions were lifted, this may not actually help trainees - the extra time allowed in work would likely mean that trainees are used to fill more rota gaps and do even more service provision.

“The managers would say ‘well you can work more than 48 hours a week now - you can work 60 hours, so you can do more on calls’.” (P5)

The main service delivery issue discussed which consultants felt to be affecting training was the issue of emergency work. As consultants noted:

“If you can do things electively, you can also do them as emergencies. Being taken out of that elective environment reduces the number of cases you can do.” (P4)

Patient care

The foremost goal of any doctor or health professional in the NHS is to deliver a safe and effective service to patients. Consultants generally felt that patient safety had not yet been compromised by the lack of experience that modern trainees had relative to them, but felt that the training restrictions caused by the EWTD may cause problems in the future if not addressed:

“At the moment there are still plenty of people who trained in an old-fashioned way… but as time goes by, I suspect there may be an impact on safety.” (P1)

Another issue raised was continuity of care, which consultants felt was important for patient safety, and which was compromised by the shift pattern encouraged by the EWTD. Without a complete understanding of a patient’s medical background, trainees are unable to provide the highest standard of care.

“… you may just be exposed to seeing patients adhoc over a couple of days, removing that continuity. Which cannot be great for patient safety… because continuity is important.” (P8)

Consultants agreed that more continuity would facilitate an improvement in patient care, as patients like to develop relationships with doctors in whom they place a lot of trust (especially surgeons who operate upon them). Continuity between the clinic and the operating theatre would allow trainees to follow the decision-making process more clearly and understand why certain decisions have been made.

“I think you should follow patients through more so you’d see them in the clinic, operate on them and follow them up in the clinic” (P5)

Professional attitudes

Consultants felt professional attitudes had been affected by the EWTD. Many of the consultants had noted a decline in what they considered to be the expected professionalism of junior doctors, which they felt was caused by the EWTD limitations.

“I don’t just go home because I’m past a certain number of hours, whereas some trainees view medicine as a non-professional job… it comes to 5 o'clock and they think ‘I’m going home’”. (P8)

Although most of the consultants felt that this was an effect of the EWTD, another consultant felt that doctors were in fact no longer treated as professionals by their managers, and as such, there was less goodwill amongst doctors to stay and work over their paid hours. The feeling of this consultant was that if some of the managerial difficulties were removed, team morale would be improved, and clinicians would be able to provide more opportunities for the junior trainees.

“They’re moving people around like units of operation time instead of treating you like professionals. People who don’t know anything about surgery are moving you around and telling you what you should and shouldn’t do… it would be much better if you had a surgeon telling you that.” (P2)

## Discussion

Many of the issues highlighted by study participants have been caused by the implementation of the EWTD, but will have no doubt been exacerbated by the current COVID-19 pandemic. Limitations of working hours have impacted on the ability of surgical trainees to gain both clinical and pastoral competencies [9], which is concerning given the increasing complexity that modern medicine presents. The relationship between trainer and trainee has also deteriorated due to the restricted hours that trainees can spend in the hospital [6]. The lack of continuity and increased prevalence of shift work because of the EWTD has increased the scope for medical errors and miscommunication [10]. Furthermore, limitations on working hours and a feeling of disempowerment among junior doctors have led to some trainees developing a “clock-on, clock-off” attitude [11].

The EWTD has resulted in an increasing proportion of the more basic work which used to be delivered by trainee surgeons now being delivered by consultants [12]. It appears that the lack of hours spent in the hospital by the juniors has had a substantial impact on this shift [6] - this, in turn, impairs the ability of consultants to fulfill service requirements of their own.

Although there is no single solution available to counteract the impact that the EWTD has had upon surgical training, study participants proposed strategies that both hospitals and trainees could adopt to improve the experiences of trainees. Simulation is a relatively new and underutilized method of improving surgical skills and receiving operative training without learning on patients [13], which enables trainees to gain experience in a controlled environment as an adjunct to ordinary training opportunities [14]. Post-CCT clinical fellowships offer the opportunity for dedicated elective surgical experience in sub-specialties such as bariatric and upper-GI surgery. Consultants and managers can improve training for trainees by designing theatre lists specifically to enable more junior surgeons the opportunity to develop their skills.

Many of the consultants made comments about trainees seeking extra training during their time off [1]. The NHS Indemnity document HSG 96/48, which lays out the legalities of which professionals are covered in which circumstances, does not specifically mention doctors staying after work voluntarily for learning opportunities. As it is unclear what the medico-legal implications would be were any mistake to occur, this may act as a deterrent to the many trainees who may wish to work longer hours [4,15].

It is the opinion of the authors of this study that further qualitative research investigating the attitudes and perceptions of other consultants around the country towards the EWTD, would be of benefit to surgical training in the UK. Furthermore, it would be beneficial to explore the perceptions of modern surgical trainees who have only ever trained under the EWTD, to discover whether they share the same concerns as the consultants in this study. If so, this would provide research-based evidence supporting the Royal College of Surgeons’ opinion that surgical training within a 48-56 hour working week is not sufficient, and - now that the UK has withdrawn from the European Union - would allow for the potential for increasing working hours to be explored.

Study limitations

Two limitations may have influenced the findings of this study. Firstly, the consultant surgeons who participated in this study were based at a tertiary trauma centre in one of the UK’s largest cities. The clinical pressures placed upon this surgical department may influence the training that trainees of this hospital receive. Repeating this study in a smaller district general hospital could yield alternative findings.

Secondly, at the time of data collection, the researcher who conducted the consultant interviews (CO) was a surgical trainee under the supervision of several study participants. Accordingly, participants may have altered their responses based upon their pre-existing relationship with the interviewer. It was not deemed feasible to use a third party to conduct the interviews due to resource limitations.

## Conclusions

From the perspective of surgical consultants, the implementation of the EWTD appears to have had an impact on surgical training, service delivery, patient care, and professionalism. Surgical consultants and trainees have a responsibility to address these issues to preserve the quality of surgical care in the future. In the meantime, newly qualified consultants should continue to receive mentorship from senior colleagues. Further research is needed to determine whether the identified themes in this study are present at other institutions and what steps can be taken to address them.
